# Aortic manipulation proxy score predicts early stroke after isolated coronary artery bypass grafting: an international multicenter cohort study

**DOI:** 10.3389/fcvm.2026.1851904

**Published:** 2026-07-22

**Authors:** Haitham Abu Khadija, Mohammad Alnees, Nizar Abu Hamdeh, Nicholay Teodorovich, Mohammad Masu'd, Ramon Cohen, Duha Najajra, Alexander Kogan, Adam Mahamid, Jebrin Alkrinawi, Eyal Nachum, Omar Ayyad, Alena Kirzhner, Kareem Ibraheem, Osama Hroub, Ehud Raanani, Ehud Karni, Yahya Z. Fraitekh, Abdalaziz Darwish, Leonid Sternik

**Affiliations:** 1 Heart Center, Kaplan Medical Center, Rehovot, Affiliated with the Hebrew University, Jerusalem, Israel; 2 Palestinian Clinical Research Center, Bethlehem, Palestine; 3 Postgraduate Medical Education, Global Clinical Scholar Research Training Program, Harvard Medical School, Boston, MA, United States; 4 Department of Internal Medicine B, Kaplan Medical Center, Rehovot, Israel; 5 Faculty of Medicine, Hebrew University of Jerusalem, Clalit Health Services, Jerusalem, Israel; 6 Department of Clinical Immunology, Allergy and AIDS, Kaplan Medical Center, Rehovot, Israel; 7 Department of Cardiac Surgery, Sheba Medical Center at Tel Hashomer, Ramat Gan, Israel; 8 Gray Faculty of Medical & Health Sciences, Tel Aviv University, Tel Aviv, Israel; 9 Department of Diabetes, Endocrinology and Metabolism, Kaplan Medical Center, Rehovot, Israel; 10 Department of Internal Medicine A, Kaplan Medical Center, Rehovot and Faculty of Medicine, Hebrew University of Jerusalem, Israel

**Keywords:** aortic manipulation, coronary artery bypass grafting, decision curve analysis, postoperative stroke, risk prediction

## Abstract

**Background:**

Stroke remains a devastating complication after coronary artery bypass grafting (CABG), often related to intraoperative aortic manipulation and embolization. However, no simple and scalable surrogate exists to approximate procedural complexity and embolic risk using routinely collected variables. We developed and evaluated the Aortic Manipulation Proxy (AMP) score for predicting 30-day stroke after isolated CABG.

**Methods:**

We conducted a retrospective international multicenter cohort study including 6,818 adults undergoing isolated CABG. The primary outcome was 30-day postoperative stroke (*n* = 133; 1.95%). The AMP score was constructed from routinely documented operative variables. Associations were assessed using Cox proportional hazards models stratified by center. Discrimination was evaluated using the area under the receiver operating characteristic curve (AUC), and incremental predictive value was examined using *Δ*AUC and decision curve analysis. Sensitivity analysis, excluding postoperative atrial fibrillation and internal validation using bootstrap resampling, was performed.

**Results:**

Higher AMP scores were associated with a graded increase in 30-day stroke risk. In center-stratified models, AMP independently predicted stroke per 1–SD increase (HR 1.46; 95% CI, 1.23–1.73; *p* < 0.001) and per 2-unit increase (HR 1.62; 95% CI, 1.30–2.01; *p* < 0.001). Kaplan–Meier analysis demonstrated stepwise separation across AMP tertiles (log-rank *p* = 0.005). Addition of AMP to a clinical model modestly improved discrimination (AUC 0.731 vs. 0.707; *Δ*AUC = 0.024; *p* = 0.021) and demonstrated improved net benefit across clinically relevant thresholds. Findings were consistent in sensitivity analyses and bootstrap validation.

**Conclusions:**

In a large multicenter CABG cohort, the AMP score was independently associated with 30-day stroke and provided incremental risk stratification beyond conventional clinical factors. As a pragmatic surrogate derived from routinely available data, the AMP score may support early postoperative risk assessment, although external validation is warranted.

## Introduction

Postoperative stroke after coronary artery bypass grafting (CABG) remains an infrequent yet devastating complication, consistently associated with excess mortality, long-term disability, prolonged hospitalization, and increased need for post-acute (1–3). Although the absolute incidence is relatively low, its clinical and economic consequences are substantial, given the high global volume of CABG procedures and the disproportionate impact a single neurologic event can exert on postoperative recovery (1, 2). From a value-based care perspective, stroke contributes disproportionately to resource utilization and costs following isolated CABG (4). Accordingly, accurate early (30-day) stroke risk stratification is essential to inform perioperative prevention strategies, optimize monitoring intensity, and support rational allocation of postoperative resources.

A dominant mechanistic pathway underlying early post-CABG stroke is cerebral embolization resulting from ascending aortic atherosclerosis and intraoperative aortic manipulation, including cannulation, cross-clamping, and side-biting clamping for proximal anastomoses, which may disrupt plaque and release particulate or thrombotic debris (2, 5, 6). Observational data support a graded relationship between the extent of aortic handling and postoperative neurologic events (5, 7). Furthermore, surgical strategies that minimize or eliminate aortic manipulation, such as anaortic or “no-touch” techniques, have been associated with lower stroke rates in large cohorts and comparative analyses (8, 9). Despite this biologic and clinical rationale, the burden of aortic manipulation has not been systematically operationalized in a simple, scalable metric applicable to routine clinical datasets.

Existing stroke risk models in CABG populations primarily rely on preoperative clinical characteristics and were not designed to capture procedural exposure to aortic manipulation (10–12). Simultaneously, many multicenter registries and real-world datasets lack consistent documentation of granular intraoperative variables, such as clamp type or number, cannulation strategy, and epiaortic imaging findings, limiting the feasibility of mechanistically aligned risk modeling at scale. Consequently, there remains a need for a pragmatic, routinely computable proxy that reflects procedural aortic manipulation burden and can be broadly implemented across heterogeneous clinical settings.

Therefore, we sought to develop and evaluate the Aortic Manipulation Proxy (AMP) score, a pragmatic and routinely computable measure designed to approximate procedural aortic manipulation burden in patients undergoing isolated CABG. In a large international multicenter cohort, we examined the association between the AMP score and 30-day postoperative stroke, assessed its independent predictive value beyond established clinical risk factors, and evaluated whether it provides incremental discrimination and clinical utility. We hypothesized that higher AMP scores would be associated with a stepwise increase in stroke risk and would meaningfully improve short-term postoperative risk stratification.

## Materials and methods

### Study design and setting

We conducted a retrospective, multicenter cohort study using consecutive adult patients who underwent coronary artery bypass grafting (CABG) between January 2010 and December 2025 at participating centers in the region. Patient-level data were extracted from institutional cardiac surgery databases and supplemented by electronic medical records review when required to confirm baseline characteristics, operative details, and postoperative outcomes. The participating centers were accounted for analytically using center stratification in time-to-event models.

The study protocol was approved by the institutional review board/ethics committee at each participating center. Given the retrospective design and use of de-identified data, the requirement for informed consent was waived in accordance with local regulations.

### Study population

**Figure 1:**
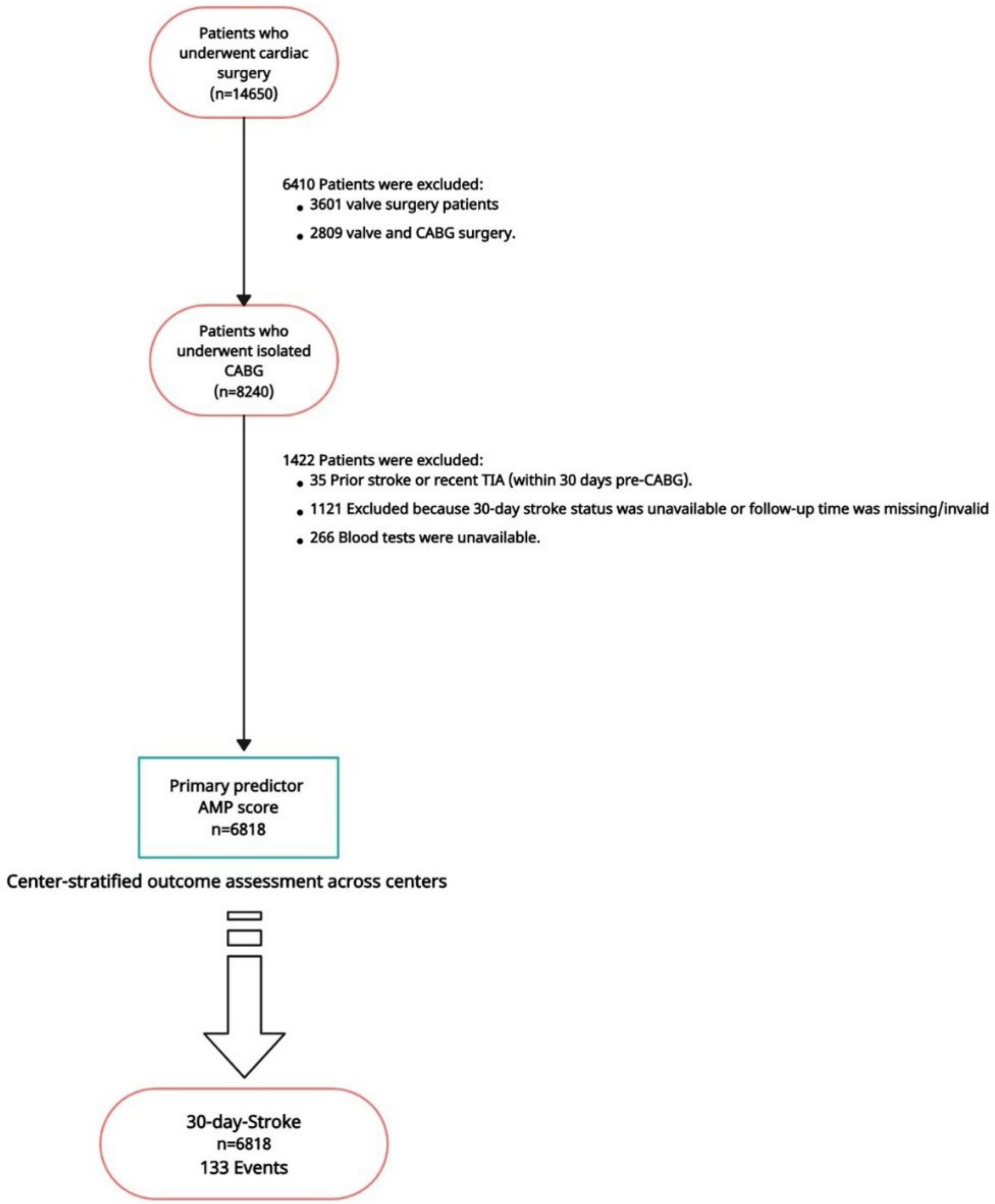
Study flow diagram of the CABG cohort and 30-Day stroke outcome.

Eligible patients were adults (≥18 years) who underwent isolated CABG during the study period. Patients undergoing valve surgery alone or combined valve-CABG procedures were excluded. Additional exclusions included patients with missing information on the primary outcome (30-day stroke status) or missing/invalid follow-up time within the 30-day postoperative window. The final analytic cohort included 6,818 patients (Figure 1). Patients with recent preoperative stroke or transient ischemic attack within 30 days before CABG and those with unavailable required laboratory data were also excluded. The vast majority of isolated CABG procedures were performed using cardiopulmonary bypass (on-pump CABG). Off-pump CABG was performed in a very small proportion of patients (<1% of the cohort), reflecting institutional practice patterns across participating centers.

### Exposure: AMP score

The primary exposure variable in this study was the Aortic Manipulation Proxy (AMP) score, a pragmatic procedural risk score designed as a surrogate construct to reflect procedural complexity and embolic vulnerability during CABG surgery. The AMP score was developed *a priori* as a pragmatic, biologically informed proxy score rather than as a regression-derived or statistically optimized prediction model. The selection and weighting of score components were guided by biological plausibility, surgical and clinical judgment, and prior evidence regarding perioperative stroke mechanisms after CABG. This approach was intended to preserve simplicity, reproducibility, and clinical scalability by relying exclusively on routinely documented variables, consistent with principles of transparent clinical prediction research and prior cardiac-surgery risk-modeling frameworks (11–13).

The score incorporates three routinely available components reflecting complementary domains of perioperative embolic risk: patient susceptibility, proximal grafting burden, and procedural complexity. Age >60 years was included as a pragmatic marker of increased vascular vulnerability and cerebrovascular susceptibility. SVG burden was included as a surrogate for increasing proximal vein-graft activity and potential exposure to aortic handling. Total graft number ≥3 was included as a marker of more extensive surgical revascularization and operative complexity. The weighting scheme was deliberately kept simple to facilitate clinical interpretability and reproducibility across centers. Accordingly, the AMP score should be interpreted as a biologically informed composite proxy of embolic exposure, procedural complexity, and patient susceptibility, rather than as a direct measure of intraoperative aortic manipulation or as a fully optimized clinical prediction model.

The score incorporates three components reflecting procedural embolic burden and patient vulnerability. First, the saphenous vein graft (SVG) count was weighted to reflect increasing aortic manipulation associated with proximal anastomoses: 0 SVGs = 0 points; 1 SVG = 2 points; 2 SVGs = 3 points; and ≥3 SVGs = 4 points. Second, the total number of grafts ≥3 was included as an indicator of overall procedural complexity (No = 0 points; Yes = 1 point). Third, age > 60 years was included to account for increased susceptibility to atheroembolism and cerebrovascular vulnerability (≤60 years = 0 points; > 60 years = 1 point). The procedural complexity component was based on the total number of grafts rather than the number of SVGs. A threshold of ≥3 grafts was selected as a pragmatic marker of more extensive surgical revascularization and a greater number of distal bypass targets (14). This component was intended to capture the overall extent of the operation, whereas the SVG component was intended to reflect proximal vein-graft burden and potential exposure to aortic manipulation. Although related, these variables capture different aspects of operative complexity and were therefore retained as separate components of the AMP score.

The total AMP score was calculated as the sum of these components, with higher scores reflecting a greater degree of procedural complexity and/or increased embolic vulnerability. For primary statistical modeling, the AMP score was analyzed both as a continuous standardized variable (per 1 standard deviation increase) and as a clinically interpretable scaled variable (per 2-unit increase). This dual modeling strategy allowed assessment of both statistical effect size comparability and practical risk gradient interpretation. Patients were categorized into tertiles of the AMP score using data-driven cut points based on the 33rd and 66th percentiles (≤2, 3–4, and >4).

### Outcome definition and follow-up

The primary outcome was 30-day postoperative stroke, defined as a clinically diagnosed ischemic or hemorrhagic stroke occurring within 30 days after CABG surgery. Stroke events were identified through institutional electronic databases and verified by detailed chart review, including confirmation by neuroimaging documentation. For statistical analyses, stroke status was coded as a binary variable.

Time-to-event was defined as the number of days from the date of surgery to the first documented stroke event. Patients who did not experience a stroke were administratively censored at 30 days. Thus, follow-up was complete and uniform for all participants through the 30-day postoperative period, ensuring consistent outcome ascertainment across centers.

Covariates were selected *a priori* based on established clinical relevance in postoperative stroke risk and on consistent availability across all participating centers (1, 2, 10, 15, 16). Significant carotid artery stenosis (CAS) was defined as ≥70% luminal narrowing based on preoperative carotid imaging and corresponding clinical documentation. Adjustment variables were chosen to capture major cardiovascular comorbidities, systemic risk factors, cardiac structural parameters, and inflammatory status, thereby reducing potential confounding in the association between the AMP score and 30-day stroke.

### Statistical analysis

Baseline characteristics were summarized using standard descriptive statistics. Continuous variables are presented as mean ± standard deviation (SD), and categorical variables as counts with corresponding percentages. Comparisons between patients who developed 30-day stroke and those who did not were performed using the two-sample t-test for continuous variables and Pearson's chi-square test or Fisher's exact test for categorical variables, as appropriate based on expected cell counts.

The primary association between the AMP score and 30-day postoperative stroke was evaluated using Cox proportional hazards regression analysis. Although follow-up was limited to 30 days, stroke was analyzed as a time-to-event outcome, with the number of days from surgery to the first documented stroke recorded for each patient. Cox proportional hazards regression was therefore used to account for differences in event timing within the follow-up period, whereas logistic regression would have considered only the occurrence of stroke without incorporating the timing of the event. To account for potential heterogeneity in baseline risk across participating centers, all Cox models were stratified by center, allowing each hospital to have its own baseline hazard function while estimating common covariate effects across centers. This approach also partially accounts for between-center heterogeneity and enhances the generalizability of the findings across different clinical settings. Hazard ratios (HRs) with 95% confidence intervals (CIs) are reported. Covariates were selected *a priori* based on established clinical relevance in postoperative stroke risk and consistent availability across participating centers (1, 2, 10, 15, 16). A crude model including the AMP score only was first constructed, followed by a multivariable-adjusted model incorporating the AMP score together with prespecified clinical covariates, including sex, hypertension, diabetes mellitus, smoking status, chronic obstructive pulmonary disease (COPD), chronic kidney disease (CKD), cardiopulmonary bypass (CPB) time, carotid artery stenosis (CAS), left ventricular end-diastolic diameter (LVEDD), interventricular septal thickness, and white blood cell (WBC) count.

Model complexity was determined with careful consideration of the 133 stroke events to preserve an appropriate events-per-variable ratio and minimize the risk of overfitting. Variables that were direct components of the AMP score were not re-entered separately into the adjusted model in order to avoid redundancy and potential collinearity. The proportional hazards assumption was evaluated using Schoenfeld residual–based testing in conjunction with visual inspection of log–log survival plots. In the presence of meaningful violations, sensitivity analyses incorporating time-varying effects would be considered.

Sensitivity analyses were performed to evaluate the robustness of the association between the AMP score and 30-day stroke. First, because preoperative atrial fibrillation was strongly associated with 30-day stroke in the baseline analysis and represents a clinically relevant baseline embolic risk factor, the multivariable center-stratified Cox models were re-estimated after adding preoperative atrial fibrillation to the adjustment set. Second, given the potential role of postoperative atrial fibrillation as a post-exposure variable that may lie on the causal pathway between procedural factors and stroke, models excluding postoperative atrial fibrillation from the adjustment set were also evaluated. Bootstrap validation using 1,000 resamples was performed to assess the stability of the sensitivity estimates. As an exploratory supplementary analysis, the association between SVG burden and 30-day stroke was evaluated using univariable Cox proportional hazards regression stratified by participating center. SVG count was categorized as 0, 1, 2, and ≥3 SVGs, with 0 SVG used as the reference category. In addition, a binary analysis comparing patients with ≥3 SVGs vs. <3 SVGs was performed to assess whether a high SVG burden was associated with increased stroke risk. Hazard ratios with 95% confidence intervals were reported.

To visualize the gradient of stroke risk across increasing AMP levels, patients were categorized into tertiles of the AMP score using data-driven cut points based on the 33rd and 66th percentiles (≤2, 3–4, and >4). Kaplan–Meier curves were generated to display the cumulative incidence of stroke during the 30-day follow-up period, with numbers at risk presented, and differences between groups were assessed using the log-rank test.

To assess incremental discrimination, two logistic regression models for 30-day stroke risk were constructed using predicted probabilities. The first model included the prespecified clinical covariates together with center indicators, while the second model incorporated all clinical covariates in addition to the standardized AMP score. Model discrimination was quantified using the area under the receiver operating characteristic curve (AUC), equivalent to the c-index. AUCs were compared using a DeLong-type test for correlated ROC curves, and the difference in AUC (*Δ*AUC) was reported with its corresponding *p*-value.

Model performance was further evaluated using apparent calibration analyses for the AMP prediction model. Predicted probabilities were derived from the logistic regression model used for discrimination and decision curve analyses, which included the AMP score, prespecified clinical covariates, and center indicators. Calibration was assessed using calibration-in-the-large, calibration slope, the Hosmer–Lemeshow goodness-of-fit test across deciles of predicted risk, and observed vs. predicted 30-day stroke rates across deciles. A calibration plot was generated to visually compare observed event rates with mean predicted risk across deciles of predicted probability. Internal validation of the Cox regression estimates was performed using bootstrap resampling with 1,000 repetitions to assess the stability of the association between AMP score and 30-day stroke.

Clinical utility was further evaluated using decision curve analysis based on predicted probabilities from both logistic models. Net benefit was calculated across clinically relevant threshold probabilities, with emphasis on the low-risk range appropriate for a relatively rare outcome such as 30-day postoperative stroke. Decision curves were plotted to compare each model against “treat all” and “treat none” strategies. Internal validation was performed using bootstrap resampling with 1,000 repetitions to assess the stability of the estimated hazard ratios. Primary analyses were conducted using complete-case data for variables included in each respective model. All statistical tests were two-sided, and a *p*-value <0.05 was considered statistically significant. All analyses were performed using Stata/BE version 17.0 (StataCorp, College Station, TX, USA), and decision curve analysis was conducted using a user-written Stata implementation.

## Results

Among patients who developed stroke (*n* = 133), the median time to event was 5 days [interquartile range (IQR) 2–10 days], with a mean of 6.8 ± 6.1 days. Most stroke events occurred early in the postoperative period, with 25% occurring within the first 2 days and 75% within 10 days. The distribution was right-skewed (skewness 1.67), reflecting a predominance of early events with fewer late occurrences up to day 30.

Among 6,818 patients undergoing CABG, 133 (1.95%) developed 30-day stroke. As shown in Table 1, patients who experienced stroke had a higher EuroSCORE II (3.22 ± 1.69 vs. 2.78 ± 2.19; *p* = 0.020) and were more likely to have hypertension (71.4% vs. 59.2%; *p* = 0.005) and chronic obstructive pulmonary disease (14.3% vs. 6.3%; *p* < 0.001). Importantly, atrial fibrillation demonstrated a strong association with postoperative stroke. Preoperative atrial fibrillation was significantly more prevalent among patients who developed stroke (23.3% vs. 9.7%; *p* < 0.001), indicating a substantial baseline embolic susceptibility. Moreover, postoperative atrial fibrillation (POAF) occurred in 20.3% of the overall cohort and was markedly more frequent in the stroke group compared with the non-stroke group (37.6% vs. 20.0%; *p* < 0.001). Echocardiographically, stroke patients demonstrated larger LVEDD (47.69 ± 8.68 mm vs. 44.82 ± 8.88 mm; *p* < 0.001) and greater septal thickness (10.61 ± 2.25 mm vs. 10.10 ± 2.37 mm; *p* = 0.014). The mean AMP score was significantly higher in the stroke group (3.38 ± 1.55 vs. 2.93 ± 1.57; *p* = 0.001).

**Table 1:** Baseline characteristics according to 30-day stroke after CABG.

Variable	Category	Overall (*n* = 6,818)	No stroke (*n* = 6,685)	Stroke (*n* = 133)	*P* value
Demographic data					
Age, y	Mean ± SD	64.66 ± 9.49	64.63 ± 9.49	66.20 ± 9.46	0.060^a^
Gender n, %	Female	1,886 (27.66%)	1,840 (27.52%)	46 (34.59%)	0.071^b^
BMI, kg/m^2^	Mean ± SD	28.29 ± 3.92	28.29 ± 3.93	28.08 ± 3.67	0.538^a^
EuroSCORE II	Mean ± SD	2.79 ± 2.18	2.78 ± 2.19	3.22 ± 1.69	0.020^a^
Medical History					
Diabetes Mellitus	Yes	2,742 (40.22%)	2,681 (40.10%)	61 (45.86%)	0.180^b^
Hypertension	Yes	4,055 (59.47%)	3,960 (59.24%)	95 (71.43%)	0.005^c^
Smoker	Yes	2,166 (31.77%)	2,115 (31.64%)	51 (38.35%)	0.100^b^
Preoperative atrial fibrillation, n (%)	Yes	680 (10.0%)	649 (9.7%)	31 (23.3%)	< 0.001^c^
History of old stroke, n (%)	Yes	545 (8.0%)	530 (7.9%)	15 (11.3%)	0.24^c^
COPD	Yes	437 (6.41%)	418 (6.25%)	19 (14.29%)	< 0.001^c^
CKD	Yes	593 (8.70%)	575 (8.60%)	18 (13.53%)	0.046^b^
Carotid artery stenosis (CAS)	Yes	667 (9.78%)	648 (9.69%)	19 (14.29%)	0.048^b^
Medication					
Insulin	Yes	283 (4.15%)	276 (4.13%)	7 (5.26%)	0.516^b^
Oral diabetic	Yes	1,865 (27.35%)	1,819 (27.21%)	46 (34.59%)	0.059^b^
Laboratory					
White blood cells (K/uL)	Mean ± SD	8.30 ± 2.89	8.29 ± 2.89	8.64 ± 2.83	0.165^a^
Platelets (K/uL)	Mean ± SD	223.50 ± 66.82	223.31 ± 66.82	232.90 ± 66.45	0.101^a^
Total cholesterol (mg/dL)	Mean ± SD	158.55 ± 49.71	158.57 ± 49.58	157.70 ± 55.91	0.841^a^
Total protein (g/dL)	Mean ± SD	6.80 ± 1.46	6.80 ± 1.47	6.78 ± 0.67	0.895^a^
Creatinine (mg/dL)	Mean ± SD	1.08 ± 0.69	1.08 ± 0.69	1.11 ± 0.52	0.571^a^
Procedural Parameters					
Number of grafts, n (%)	1	1,044 (15.3%)	1,026 (98.28%)	18 (1.72%)	< 0.001^b^
	2	2,188 (32.1%)	2,147 (98.13%)	41 (1.87%)	
	3	2,488 (36.5%)	2,441 (98.11%)	47 (1.89%)	
	4	996 (14.6%)	984 (98.80%)	12 (1.20%)	
	5	102 (1.5%)	87 (85.29%)	15 (14.71%)	
LIMA use, n (%)		6,582 (96.54%)	6,453 (96.53%)	129 (96.99%)	0.772^c^
RIMA use, n (%)		1,969 (28.88%)	1,929 (28.86%)	40 (30.08%)	0.945^b^
SVG use, n (%)		4,913 (72.06%)	4,805 (71.88%)	108 (81.20%)	0.018^c^
SVG count	Mean ± SD	1.00 ± 0.79	0.99 ± 0.79	1.26 ± 0.87	< 0.001^a^
Radial artery use, n (%)		721 (10.57%)	702 (10.50%)	19 (14.29%)	0.160^b^
Cardiopulmonary Bypass time (min)	Mean ± SD	90.5 ± 35.9	90.3 ± 35.9	97.3 ± 36.5	0.028^a^
Aortic cross-clamp time (min)	Mean ± SD	58.3 ± 26.0	58.3 ± 26.0	60.9 ± 24.4	0.249^a^
Postoperative atrial fibrillation, n (%)		1,387 (20.34%)	1,337 (20.00%)	50 (37.59%)	< 0.001^c^
Echocardiography					
Septum thickness (mm)	Mean ± SD	10.11 ± 2.37	10.10 ± 2.37	10.61 ± 2.25	0.0139^a^
LVEDD, mm	Mean ± SD	44.87 ± 8.89	44.82 ± 8.88	47.69 ± 8.68	0.0002^a^
LVEF%	Mean ± SD	50.73 ± 10.93	50.76 ± 10.94	49.19 ± 10.16	0.100^a^
Primary exposure					
AMP Score	Mean ± SD	2.94 ± 1.57	2.93 ± 1.57	3.38 ± 1.55	0.001^a^

BMI, body mass index; CABG, coronary artery bypass grafting; COPD, chronic obstructive pulmonary disease; CKD, chronic kidney disease; CAS, carotid artery stenosis; WBC, white blood cell count; PLT, platelet count; LIMA, left internal mammary artery; RIMA, right internal mammary artery; SVG, saphenous vein graft; LVEDD, left ventricular end-diastolic diameter; LVEF, left ventricular ejection fraction; AMP Score, aortic manipulation proxy.

a Two-sample t test.‏

b Pearson chi-square test.‏

c Fisher's exact test.

In univariable Cox regression analysis (Table 2), hypertension (HR 1.71, 95% CI 1.18–2.50; *p* = 0.005), chronic obstructive pulmonary disease (HR 2.47, 95% CI 1.52–4.01; *p* < 0.001), and chronic kidney disease (HR 1.65, 95% CI 1.01–2.72; *p* = 0.047) were associated with increased 30-day stroke risk. Echocardiographic parameters were also significant predictors, including LVEDD (HR 1.04 per mm, 95% CI 1.02–1.06; *p* < 0.001) and septal thickness (HR 1.09 per mm, 95% CI 1.02–1.17; *p* = 0.014).

**Table 2:** Univariable Cox regression analysis for 30-day stroke after CABG (*N* = 6,818, 133 events).

Variable	Category	Stroke *N* = 6,818 133 events	
		HR (95% CI)	*P* value
Demographic data			
Age, y	per 1-year increase	1.02 (1.00–1.04)	0.061
Gender n, %	Female vs. male	1.39 (0.97–1.98)	0.072
BMI, kg/m^2^	per 1-unit increase	0.99 (0.94–1.03)	0.536
EuroSCORE II	per 1-unit increase	1.08 (1.01–1.16)	0.020
Medical History			
Diabetes Mellitus	Yes, vs. No	1.26 (0.90–1.77)	0.182
Hypertension	Yes, vs. No	1.71 (1.18–2.50)	0.005
Smoker	Yes, vs. No	1.34 (0.95–1.90)	0.100
COPD	Yes, vs. No	2.47 (1.52–4.01)	<0.001
CKD	Yes, vs. No	1.65 (1.01–2.72)	0.047
Carotid artery stenosis (CAS)	Yes, vs. No	1.55 (0.95–2.52)	0.078
Medication			
Insulin	Yes, vs. No	1.29 (0.60–2.75)	0.518
Oral diabetic	Yes, vs. No	1.41 (0.98–2.01)	0.061
Laboratory			
White blood cells (K/uL)	per 1-unit increase	1.03 (0.99–1.08)	0.154
Platelets (K/uL)	per 10-unit increase	1.02 (1.00–1.04)	0.099
Total cholesterol (mg/dL)	per 10-unit increase	1.00 (0.96–1.03)	0.848
Total protein (g/dL)	per 1-unit increase	0.99 (0.83–1.18)	0.898
Creatinine (mg/dL)	per 1-unit increase	1.06 (0.86–1.31)	0.571
Echocardiography			
Septum thickness (mm)	per 1-mm increase	1.09 (1.02–1.17)	0.014
LVEDD, mm	per 1-mm increase	1.04 (1.02–1.06)	<0.001
LVEF%	per 1% increase	0.98 (0.97–1.00)	0.062
Cardiopulmonary Bypass time (min)	per 10-min increase	1.05 (1.01–1.09)	0.022
Aortic cross-clamp time (min)	per 10-min increase	1.02 (0.98–1.06)	0.25
Postoperative atrial fibrillation, n (%)	Yes, vs. No	1.02 (0.98–1.06)	0.32

BMI, body mass index; COPD, chronic obstructive pulmonary disease; CKD, chronic kidney disease; LVEDD, left ventricular end-diastolic diameter; LVEF, left ventricular ejection fraction; WBC, white blood cell count; HR, hazard ratio; CI, confidence interval.

**Figure 2:**
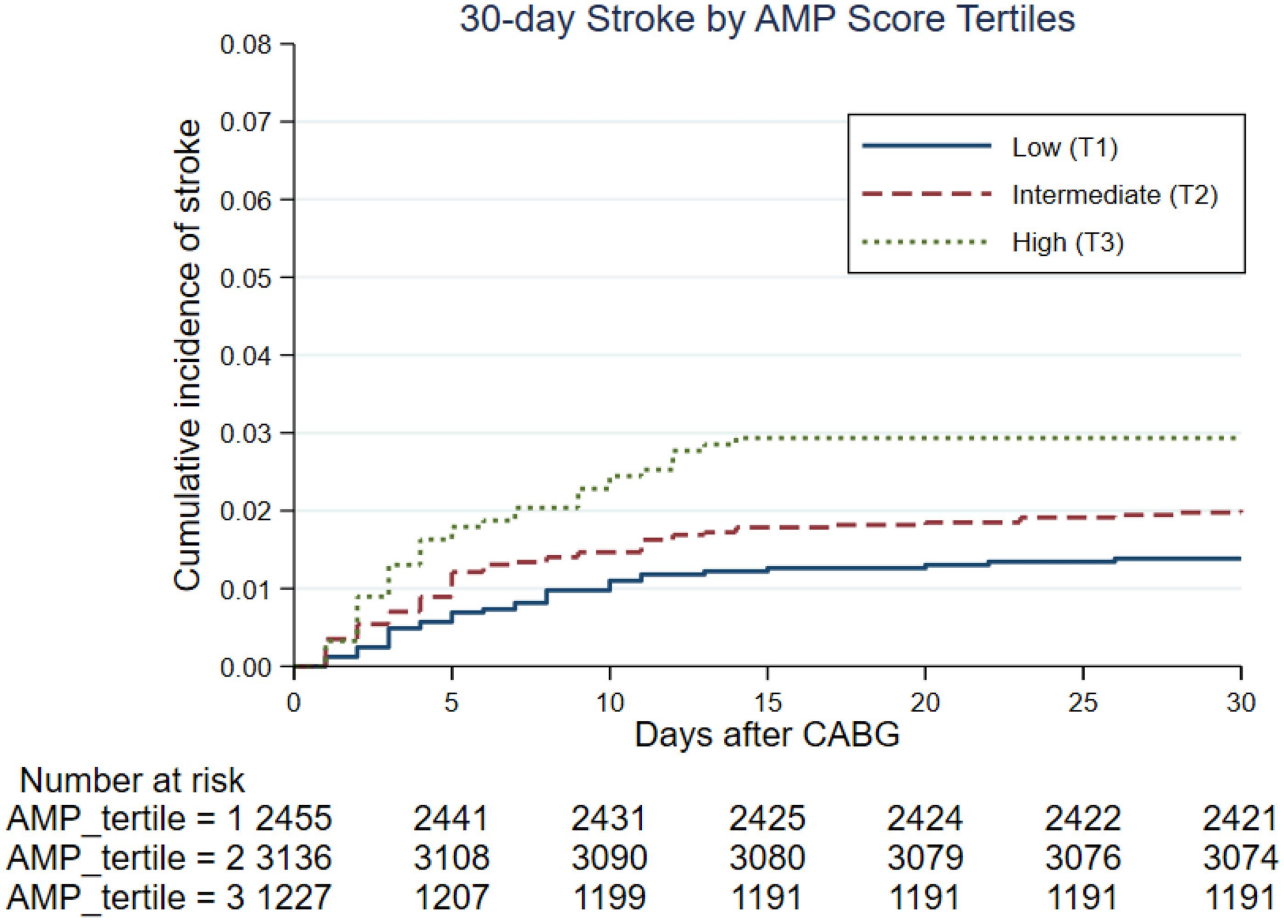
Kaplan–Meier estimates of 30-Day stroke according to AMP score tertiles.

Kaplan–Meier analysis demonstrated a stepwise increase in 30-day stroke incidence across AMP score tertiles (Figure 2). Patients in the highest tertile (T3) exhibited the greatest cumulative incidence of stroke, followed by the intermediate (T2) and lowest tertile (T1). At 30 days, the cumulative incidence of stroke was approximately 2.9% in T3, compared with 2.0% in T2 and 1.4% in T1, demonstrating a clear risk gradient across increasing AMP score categories. Log-rank testing confirmed a statistically significant difference in stroke risk between tertiles (*χ*^2^ = 10.43, *p* = 0.0054), supporting the discriminatory capacity of the AMP score in stratifying early postoperative stroke risk. The number at risk for each tertile at prespecified time points is shown below the graph.

In stratified Cox regression analyses accounting for center-level effects, the AMP score was significantly associated with 30-day stroke risk (Table 3). In crude models stratified by participating centers, each 1-standard deviation (SD) increase in the AMP score was associated with a 45% higher hazard of 30-day stroke (HR 1.45, 95% CI 1.23–1.72; *p* < 0.001). Similarly, each 2-unit increase in the AMP score was associated with a 61% increased hazard of stroke (HR 1.61, 95% CI 1.30–2.00; *p* < 0.001).

**Table 3:** Association between AMP score and 30-Day stroke (stratified by center).

AMP Score	Stroke *N* = 6,818; events = 133	
	HR (95% CI)	*P* value
*Crude (stratified by Centers)*		
AMP Score per 1-SD increase	1.45 (1.23–1.72)	<0.001
AMP Score per 2-unit increase	1.61 (1.30–2.00)	<0.001
Adjusted (stratified by Centers)*		
AMP Score per 1-SD increase	1.46 (1.23–1.73)	<0.001
AMP Score per 2- unit increase	1.62 (1.30–2.01)	<0.001
Bootstrap Adjusted (stratified by Centers)*		
AMP Score per 1-SD increase	1.46 (1.24–1.72)	<0.001
AMP Score per 2- unit increase	1.62 (1.31–1.99)	<0.001

Adjusted for sex, hypertension, diabetes mellitus, smoking status, chronic obstructive pulmonary disease (COPD), chronic kidney disease (CKD), cardiopulmonary bypass (CPB) time, carotid artery stenosis (CAS), left ventricular end-diastolic diameter (LVEDD), interventricular septal thickness, and white blood cell (WBC) count. All Cox models were stratified by participating center.

After adjustment for clinically relevant covariates—including sex, hypertension, diabetes mellitus, smoking status, chronic obstructive pulmonary disease, chronic kidney disease, carotid artery stenosis, left ventricular end-diastolic diameter, interventricular septal thickness, and white blood cell count—the association remained consistent. In the fully adjusted model, each 1-SD increase in the AMP score was associated with a 46% higher hazard of 30-day stroke (HR 1.46, 95% CI 1.23–1.73; *p* < 0.001), and each 2-unit increase conferred a 62% increased hazard (HR 1.62, 95% CI 1.30–2.01; *p* < 0.001).

Bootstrap validation yielded similar estimates, confirming the stability and robustness of the association between the AMP score and 30-day stroke. The complete multivariable Cox regression models are presented in Supplementary eTable 1 and eTable 2 (Supplement 1).

**Figure 3:**
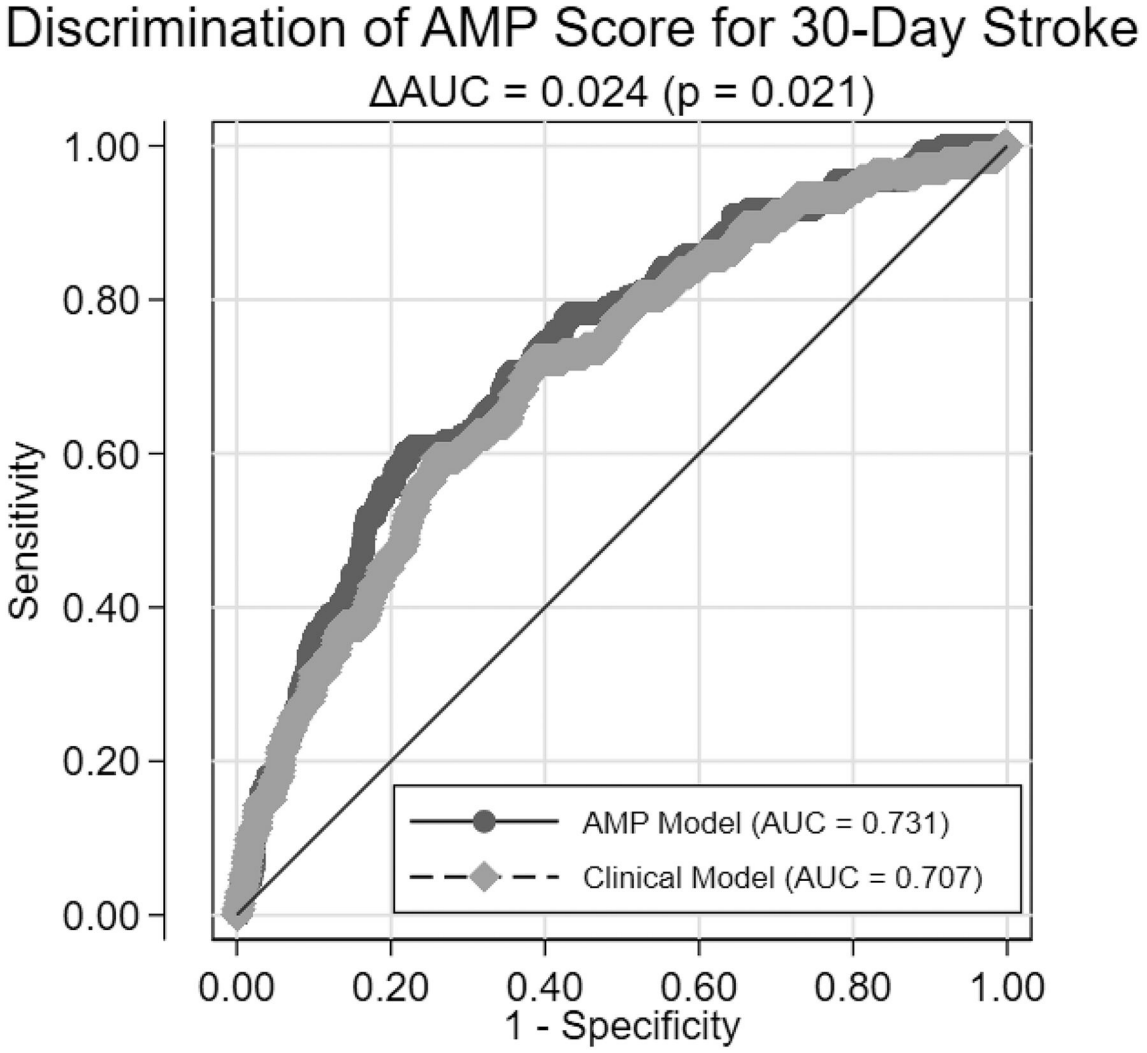
Discriminative performance of the AMP score for 30-Day stroke. Receiver operating characteristic (ROC) curves comparing the discriminative performance of the AMP score–based model and the clinical model for prediction of 30-day stroke. The AMP model demonstrated superior discrimination (AUC 0.731; 95% CI approximately 0.69–0.77) compared with the clinical model (AUC 0.707; 95% CI approximately 0.66–0.75), with a statistically significant improvement in discrimination (ΔAUC = 0.024; *p* = 0.021).

The AMP model demonstrated superior discriminative performance for the prediction of 30-day stroke compared with the clinical model. As shown in Figure 3, the area under the receiver operating characteristic curve (AUC) was significantly higher for the AMP model (AUC 0.731) than for the clinical model (AUC 0.707), corresponding to a statistically significant improvement in discrimination (*Δ*AUC = 0.024; *p* = 0.021).

**Figure 4:**
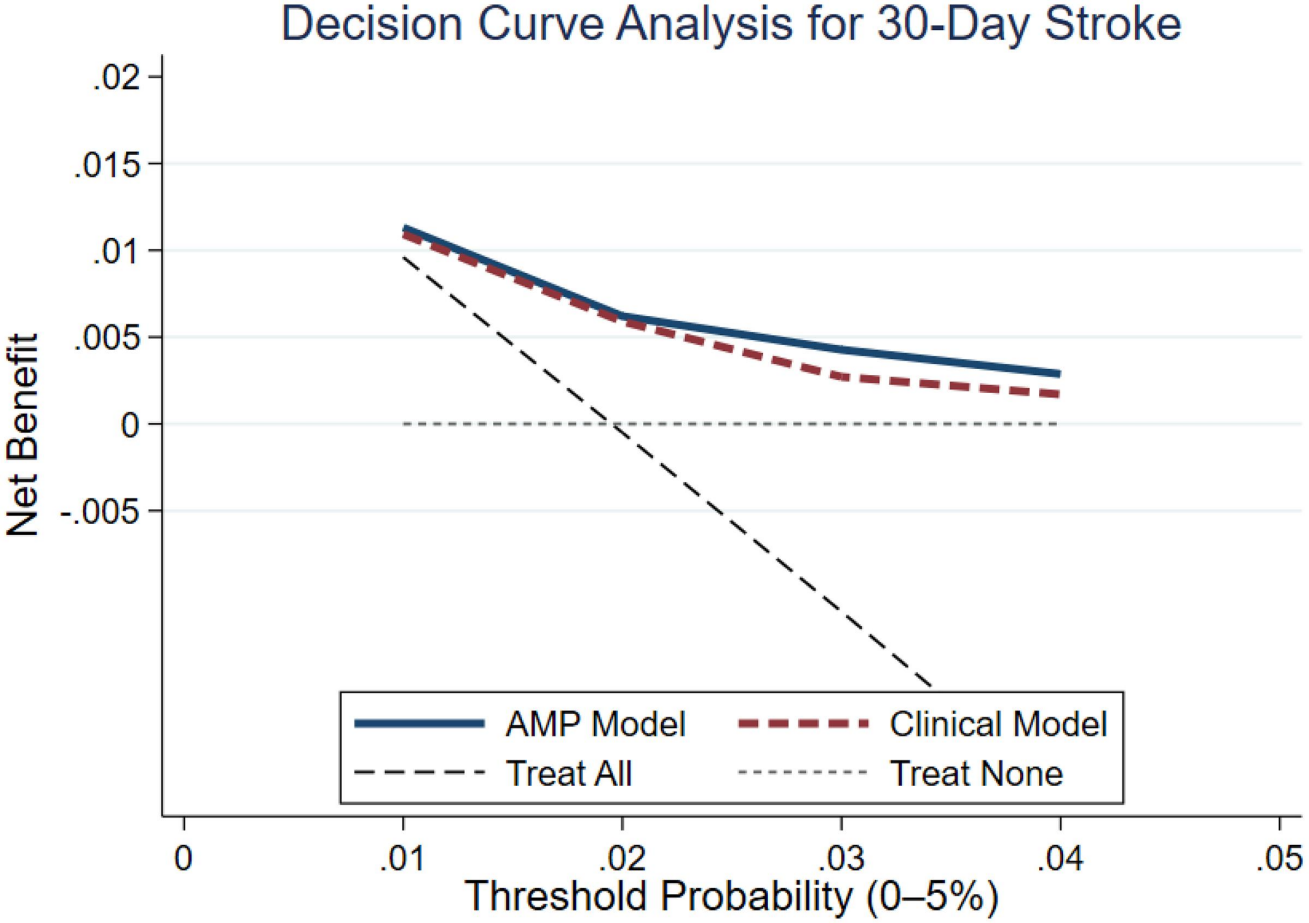
Decision curve analysis for clinical utility of the AMP score. Decision curve analysis showing the net clinical benefit of the AMP model relative to the clinical model, treat-all, and treat-none strategies at clinically relevant threshold probabilities (0%–5%). The AMP model consistently showed a higher net benefit than the clinical model across clinically meaningful decision thresholds.

Beyond discrimination, assessment of clinical utility using decision curve analysis further supported the incremental value of the AMP score. As illustrated in Figure 4, the AMP model consistently provided a greater net benefit across clinically relevant threshold probabilities (1%–5%) than the clinical model, as well as the treat-all and treat-none strategies. These findings indicate that incorporation of the AMP score may enhance individualized perioperative risk stratification and clinical decision-making for prevention of postoperative stroke.

In sensitivity analysis excluding postoperative atrial fibrillation from the adjustment model, the association between the AMP score and 30-day stroke remained consistent. The AMP score continued to demonstrate a significant association with stroke risk both per 1-standard deviation increase (HR 1.46, 95% CI 1.23–1.73; *p* < 0.001) and per 2-unit increase (HR 1.62, 95% CI 1.30–2.01; *p* < 0.001). Bootstrap validation yielded similar estimates (Supplementary eTable 3 in Supplement 1), supporting the robustness and stability of the findings.

In sensitivity analysis incorporating preoperative atrial fibrillation into the adjusted center-stratified Cox model, the association between the AMP score and 30-day stroke remained robust (Supplementary eTable 4). After adjustment for preoperative atrial fibrillation and all prespecified covariates, each 1-SD increase in AMP score was associated with a higher hazard of 30-day stroke (HR 1.47; 95% CI, 1.24–1.75; *p* < 0.001), and each 2-unit increase in AMP score showed a similar association (HR 1.63; 95% CI, 1.31–2.03; *p* < 0.001). Preoperative atrial fibrillation was independently associated with 30-day stroke (HR 2.87; 95% CI, 1.92–4.30; *p* < 0.001). Bootstrap validation yielded consistent estimates, with AMP remaining significantly associated with stroke per 1-SD increase (HR 1.47; 95% CI, 1.25–1.73; *p* < 0.001) and per 2-unit increase (HR 1.63; 95% CI, 1.32–2.01; *p* < 0.001), supporting the stability of the findings.

In an exploratory univariable analysis evaluating age by decade-based categories, stroke incidence increased numerically across age strata, from 1.54% among patients aged <60 years to 3.21% among patients aged ≥80 years (Supplementary eTable 5). In center-stratified Cox regression using patients aged <60 years as the reference group, the hazard ratios were 1.14 (95% CI, 0.72–1.81; *p* = 0.567) for ages 60–69 years, 1.63 (95% CI, 1.02–2.60; *p* = 0.040) for ages 70–79 years, and 1.86 (95% CI, 0.91–3.82; *p* = 0.091) for ages ≥80 years. The overall model did not reach statistical significance (likelihood-ratio *p* = 0.0997), and the ≥80-year category included relatively few patients and events.

In exploratory analysis of SVG burden, 30-day stroke incidence increased progressively across SVG count categories, from 1.31% among patients with 0 SVGs to 1.86% with 1 SVG, 2.47% with 2 SVGs, and 5.33% among patients with ≥3 SVGs (Supplementary eTable 6). In center-stratified univariable Cox regression, increasing SVG count was associated with higher stroke risk compared with 0 SVGs: HR 1.72 (95% CI, 1.07–2.77; *p* = 0.025) for 1 SVG, HR 2.26 (95% CI, 1.35–3.78; *p* = 0.002) for 2 SVGs, and HR 3.64 (95% CI, 1.83–7.26; *p* < 0.001) for ≥3 SVGs. In the binary analysis, ≥3 SVGs was also associated with increased 30-day stroke risk compared with <3 SVGs (HR 2.32; 95% CI, 1.28–4.21; *p* = 0.006).

Calibration analysis of the AMP prediction model demonstrated acceptable apparent agreement between predicted and observed 30-day stroke risk (Supplementary eTable 7 and Supplementary eFigure 1). The Hosmer–Lemeshow goodness-of-fit test did not indicate significant lack of fit (*χ*^2^ = 8.23; *p* = 0.4117). Calibration-in-the-large showed no evidence of systematic overprediction or underprediction, and the apparent calibration slope was 1.00. Across deciles of predicted risk, observed event rates generally followed the expected increase in predicted risk, ranging from 0.29% in the lowest decile to 6.61% in the highest decile. These findings support acceptable apparent calibration of the AMP prediction model within the present cohort, while recognizing that external calibration remains necessary.

## Discussion

The present study introduces the AMP score as a pragmatic and scalable surrogate construct reflecting procedural complexity and embolic vulnerability in patients undergoing CABG. Although termed the Aortic Manipulation Proxy (AMP) score, the score does not directly quantify intraoperative aortic manipulation. Instead, it combines variables reflecting potential exposure to aortic handling with factors associated with patient vulnerability and procedural complexity. The AMP score was developed using biological rationale, surgical considerations, and evidence from the existing literature rather than statistical optimization. Its primary objective was to provide a simple and widely applicable proxy measure using variables routinely available in clinical practice, particularly in real-world datasets where detailed intraoperative information is often lacking. Accordingly, AMP should be interpreted as a pragmatic surrogate of perioperative embolic risk rather than as a direct measurement of aortic manipulation alone. Despite this inherent limitation, the observed graded association between AMP score and 30-day stroke, supported by consistent findings across tertiles, adjusted models, and sensitivity analyses, supports its potential value as a risk stratification tool that warrants further external validation and prospective evaluation.

An important methodological aspect of the present study is that the AMP score was intentionally designed as a pragmatic *a priori* construct, not as a regression-derived or statistically optimized risk model. This distinction is central to its interpretation. The goal was to create a simple and reproducible score that could be computed from routinely available CABG variables in real-world datasets where detailed intraoperative information regarding clamp strategy, cannulation technique, epiaortic imaging, and aortic atherosclerotic burden is often unavailable. This approach is consistent with transparent clinical prediction principles and with cardiac-surgery risk-modeling frameworks that emphasize the use of clinically meaningful and consistently captured variables (11–13).

Although the score weights were not statistically derived, the AMP score underwent a comprehensive post-construction statistical evaluation. The association between AMP and 30-day stroke remained consistent when modeled per 1-SD increase and per 2-unit increase, and persisted after adjustment for prespecified clinical covariates in center-stratified Cox models. The stepwise separation across AMP tertiles further supported a graded risk relationship rather than an isolated threshold effect. Beyond association testing, the AMP score modestly but significantly improved discrimination when added to the clinical model, demonstrated higher net benefit across clinically relevant low-risk thresholds on decision curve analysis, and showed stable estimates during bootstrap internal validation.

These findings support the prognostic relevance of the AMP construct while also defining its limitations. AMP should not be interpreted as a direct measurement of aortic manipulation or as a definitive prediction model ready for unqualified clinical implementation. Rather, it should be viewed as an initial pragmatic proxy score that links surgical mechanism, patient vulnerability, and routinely captured operative data. Future studies should externally validate the score, formally assess calibration, explore alternative weighting strategies, and determine whether adding granular intraoperative variables such as clamp number, clamp strategy, cannulation technique, epiaortic ultrasound findings, and anaortic approaches can further improve predictive performance.

The additional sensitivity analysis including preoperative atrial fibrillation further supports the robustness of the AMP–stroke association. Although preoperative atrial fibrillation was independently associated with 30-day stroke, adjustment for this baseline embolic risk factor did not attenuate the association between AMP score and stroke. This suggests that the prognostic value of AMP is not simply explained by baseline atrial arrhythmia burden, but may reflect additional procedural complexity and embolic vulnerability captured by the score. The exploratory SVG analysis supports the biological plausibility of the AMP score by demonstrating a graded increase in 30-day stroke risk with increasing SVG burden. This finding is consistent with the concept that a greater number of SVGs may reflect higher proximal vein-graft burden and greater opportunity for aortic handling. However, this analysis should not be interpreted as redefining the procedural complexity component of the AMP score. In the AMP construct, SVG count and total graft number were intentionally retained as related but distinct domains: SVG count reflects proximal vein-graft burden, whereas total graft number ≥3 reflects the overall extent of surgical revascularization. Because the AMP score was developed *a priori* as a pragmatic proxy rather than as a *post hoc* regression-derived weighted model, the original score structure was retained.

Although the incremental improvement in discrimination was modest (*Δ*AUC = 0.024), this degree of gain is consistent with prior clinical risk modeling studies in relatively low-incidence outcomes and should be interpreted cautiously when considering potential clinical application. Importantly, the robustness of the findings was supported by sensitivity analyses excluding postoperative atrial fibrillation, addressing potential concerns regarding over-adjustment of post-exposure variables, as well as by bootstrap validation, which demonstrated stability of effect estimates and minimized concerns regarding overfitting.

The additional calibration analysis provides a more complete assessment of AMP model performance beyond discrimination alone. The model demonstrated acceptable apparent calibration, with no significant lack of fit on Hosmer–Lemeshow testing and close agreement between observed and predicted risk across most deciles. However, this calibration assessment was performed within the derivation cohort and should therefore be interpreted as apparent internal calibration rather than evidence of external transportability. Future studies should externally validate the AMP model and assess calibration performance in independent CABG populations before clinical implementation as an individualized risk prediction tool.

Furthermore, the use of center-stratified Cox regression models allowed for accommodation of heterogeneity in baseline risk across participating institutions, thereby supporting the consistency of the observed association across participating centers. This methodological approach does not replace formal external validation, which remains necessary to confirm calibration, transportability, and clinical utility in independent cohorts.

From a clinical perspective, the AMP score is not intended to replace existing risk models or to guide specific intraoperative decisions in isolation, but rather to provide a simple and reproducible framework for early postoperative risk stratification. In settings where detailed intraoperative variables are unavailable, such a pragmatic proxy may help identify patients at higher estimated risk who could be considered for closer monitoring or individualized perioperative assessment, although its role in guiding management requires prospective validation.

These findings are biologically and surgically plausible because the AMP components map to embolic opportunity and host susceptibility: higher SVG counts typically imply more proximal aortic anastomoses (often requiring side-biting clamping), greater total graft number reflects procedural complexity and a longer “exposure window,” and older age is associated with a higher prevalence of aortic atheroma and reduced cerebrovascular reserve. Although vascular risk increases progressively with advancing age, the age component of the AMP score was intended as a simple pragmatic marker of embolic vulnerability rather than a detailed measure of age-related atherosclerotic burden. The >60-year threshold was selected *a priori* to preserve simplicity, interpretability, and applicability across clinical settings. Exploratory analysis of decade-based age categories (<60, 60–69, 70–79, and ≥80 years) demonstrated a numerical increase in stroke incidence with advancing age, but the overall center-stratified Cox model did not reach statistical significance, and the ≥80-year subgroup included relatively few patients and events. Therefore, the available data did not support *post hoc* introduction of additional age strata or age-specific weights into the AMP score. Retaining the original >60-year component preserves the prespecified and pragmatic nature of the score, while future validation cohorts with larger numbers of very elderly patients may further evaluate whether alternative age-weighting strategies improve risk stratification. Increasing degrees of aortic manipulation have been associated with higher neurological event rates after CABG, while avoidance of aortic clamping and other approaches that minimize ascending aortic handling have been linked to lower stroke risk (5, 8, 9). Additional evidence from epiaortic ultrasound studies and meta-analyses supports the concept that identifying and avoiding severe ascending aortic atheroma may reduce perioperative cerebral embolization and stroke risk, further reinforcing the mechanistic plausibility of an aortic manipulation burden construct (17–19). Importantly, AMP should be interpreted as a pragmatic proxy of “aortic manipulation burden” rather than a direct causal measurement of clamp strategy or cannulation technique.

Our observed 30-day stroke incidence (1.95%) aligns with the range reported in prior CABG literature. Mérie et al. reported an approximately 2.0% 30-day stroke rate, while Roach et al. documented major neurologic events after CABG and highlighted their substantial prognostic impact. Shroyer et al. further emphasized the importance of early postoperative stroke risk assessment by incorporating stroke within the STS 30-day operative morbidity framework (2, 15, 16, 20, 21). Female sex emerged as an independent predictor of 30-day stroke in the adjusted analysis. This finding is consistent with prior literature demonstrating sex-related differences in outcomes after CABG. Dumitriu LaGrange et al. reported higher rates of postoperative stroke and short-term adverse outcomes among women undergoing CABG (22), while Gaudino et al. demonstrated a higher risk of major adverse cardiovascular and cerebrovascular events among women compared with men after CABG (23). Potential explanations include differences in baseline risk profile, vascular anatomy, conduit selection, and other factors not fully captured by measured covariates. However, because the present study was not designed to investigate sex-specific mechanisms, this association should be interpreted cautiously and warrants further investigation. Prior surgical literature also supports the mechanistic link between aortic handling and stroke reduction: Moss et al., Edelman et al.; and Zhao et al. each reported lower neurologic events with “no-touch/anaortic” or reduced aortic manipulation approaches (8, 9, 24–29). What differentiates the current work is that AMP is computable from routinely recorded CABG variables and demonstrates a consistent signal across centers using center-stratified time-to-event modeling, supporting scalability in real-world datasets where granular aortic handling data are often unavailable. The novelty of our study is the development and multicenter evaluation of a pragmatic Aortic Manipulation Proxy (AMP) score that can be computed entirely from routinely documented CABG variables, enabling a standardized way to approximate procedural aortic manipulation burden at scale. Importantly, this introduces a potential translational framework by bridging surgical mechanisms and real-world data. This approach may support future efforts to implement early stroke risk stratification even when granular intraoperative fields are unavailable. It may also facilitate harmonized benchmarking of stroke risk and procedural “aortic handling burden” across institutions, pending further validation. In addition, it provides a practical foundation for quality-improvement and future implementation studies designed to test whether AMP-guided perioperative pathways and aortic strategies can reduce neurologic events.

Although direct intraoperative measures of aortic manipulation were unavailable, the observed perioperative patterns provide indirect support for the construct plausibility of the AMP score. In the present cohort, patients who developed 30-day stroke had greater SVG use, higher SVG count, longer cardiopulmonary bypass time, and a higher mean AMP score, whereas the cohort was composed almost entirely of on-pump CABG procedures. These findings are clinically coherent with the concept that increasing proximal grafting burden and procedural complexity may identify patients with greater opportunity for embolic exposure, even when the exact number of clamp applications, clamping strategy, cannulation approach, or aortic plaque burden cannot be measured directly. Moreover, the association between AMP and stroke was not limited to a single analytic approach; it was supported by a graded risk pattern across tertiles, persisted in center-stratified adjusted Cox models, and remained stable in sensitivity and bootstrap analyses. Therefore, the present findings support AMP as a pragmatic prognostic proxy reflecting procedural complexity and embolic vulnerability, while not establishing it as a direct quantitative measure of intraoperative aortic manipulation.

Clinically, AMP may be considered as a tool for risk stratification and operational triage rather than as a stand-alone directive for specific therapies. For example, Charlesworth et al. and Shroyer et al. developed CABG stroke-risk tools primarily from preoperative/clinical predictors; AMP can complement such approaches by adding a simple procedural-proxy layer that may help teams anticipate neurologic risk and inform aortic strategy discussions (e.g., selective epiaortic assessment, consideration of reduced-clamp approaches where appropriate) (10, 15). This framing is consistent with perioperative stroke-prevention literature emphasizing structured risk recognition, tailored intraoperative planning, and postoperative surveillance as key components of neurologic risk mitigation in cardiac surgery rather than one-size-fits-all treatment directives (21, 30).

### Strengths and limitations

This study has several strengths. First, the multicenter design, combined with center-stratified Cox modeling, accounts for heterogeneity in baseline risk across institutions, thereby enhancing the generalizability of the findings across diverse clinical settings. Second, the consistency of results across complementary analytical approaches—including Kaplan–Meier risk gradients, adjusted time-to-event models, incremental discrimination analyses, and decision curve analysis—supports the robustness of the observed associations. Third, the AMP score was derived entirely from routinely documented variables, enabling broad applicability and scalability in real-world datasets without reliance on detailed intraoperative documentation. Finally, the stability of the findings was reinforced through sensitivity analyses and bootstrap validation, reducing concerns regarding model overfitting and supporting the reliability of the estimated effects.

Several limitations warrant consideration. First, the retrospective design introduces the possibility of residual confounding and variation in practice patterns across centers. Second, although stroke events were clinically adjudicated, differences in diagnostic thresholds and imaging practices between institutions may have influenced outcome ascertainment. Third, although direct intraoperative measures of aortic manipulation were not available, the study included several routinely documented operative variables that partially characterize the procedural context relevant to the AMP construct, including SVG use, SVG count, total graft number, cardiopulmonary bypass time, aortic cross-clamp time, and operative strategy. The observed perioperative patterns were clinically coherent with the proposed construct, as patients who developed 30-day stroke had greater SVG use, higher SVG count, longer cardiopulmonary bypass time, and higher AMP scores. These findings support the interpretation of AMP as a pragmatic surrogate reflecting proximal grafting burden, procedural complexity, and perioperative embolic vulnerability using routinely available clinical and operative variables. Nevertheless, the absence of granular intraoperative variables remains an important limitation. Detailed measures such as side-biting clamp use, number of clamp applications, single-versus multiple-clamp strategies, no-touch or anaortic techniques, epiaortic ultrasound findings, cannulation strategy, and direct assessment of ascending aortic atherosclerotic burden were not available within the registry. In addition, SVG count should be viewed as a surrogate marker rather than a direct measure of aortic manipulation, because multiple proximal anastomoses may be constructed during a single side-biting clamp application and clamping strategies may vary across surgeons and institutions. Therefore, although the available operative variables provide indirect construct support, they cannot directly quantify aortic manipulation in individual patients, and the present study cannot directly validate AMP against measured aortic manipulation burden. Because off-pump CABG accounted for less than 1% of the study population, the present findings primarily reflect patients undergoing conventional on-pump CABG and may not be directly generalizable to predominantly off-pump or anaortic revascularization strategies. Future studies incorporating granular intraoperative data are needed to determine how closely AMP correlates with measured aortic handling and whether refinement using direct operative variables improves predictive performance, calibration, and clinical utility.

In addition, the incremental improvement in discrimination, while statistically significant, was modest, and therefore the clinical impact of the score should be interpreted cautiously. Furthermore, although internal validation using bootstrap resampling and sensitivity analyses supported the robustness of the findings, external validation in independent cohorts is required to confirm calibration, transportability, and clinical utility across different healthcare settings.

The AMP score should be considered an initial pragmatic proxy construct rather than a definitive or fully optimized risk model. Future studies should externally validate the score, evaluate alternative weighting strategies, and determine whether the incorporation of more granular intraoperative variables, such as side-biting clamp use, clamp number, cannulation strategy, epiaortic imaging findings, and anaortic techniques, can improve predictive performance. Prospective studies are also needed to determine whether AMP-guided risk stratification can improve perioperative decision-making or clinical outcomes.

## Conclusion

In a large multicenter cohort of 6,818 patients undergoing isolated CABG, the AMP score demonstrated a graded association with 30-day stroke and remained independently associated with stroke risk in center-stratified models. Although the incremental improvement in discrimination was modest, the AMP score provided additional risk stratification beyond conventional clinical factors and showed favorable clinical utility in decision curve analysis. These findings support the role of the AMP score as a pragmatic and scalable tool for early postoperative stroke risk stratification, particularly in real-world settings where detailed intraoperative data are not routinely available. Further external validation and prospective evaluation are warranted to define its role in clinical decision-making.

## Data Availability

The datasets generated and analyzed during the current study are not publicly available because of patient confidentiality and institutional ethical restrictions. Data may be available from the corresponding author upon reasonable request and subject to applicable institutional approvals.
