# Supplementary material for: Aortic manipulation proxy score predicts early stroke after isolated coronary artery bypass grafting: an international multicenter cohort study
Source: Front Cardiovasc Med. 2026 Jul 22;13:1851904. doi: 10.3389/fcvm.2026.1851904 (PMC13437369; doi:10.3389/fcvm.2026.1851904)
Supplement: Supplementary file 1 [file Datasheet1.docx]

**Aortic Manipulation Proxy (AMP) Score Predicts Early Stroke After Isolated CABG: International Multicenter Cohort Study**

**Supplement 1**

**eTable 1. Full Multivariable Cox Model Including AMP Score (Per 1-SD Increase)**

**eTable 2. Full Multivariable Cox Model Including AMP Score (Per 1-SD Increase)**

**eTable 3. Sensitivity Analysis Excluding Postoperative Atrial Fibrillation**

**eTable 4. Sensitivity Analysis Including Preoperative Atrial Fibrillation
Stratified by participating centers**

**eTable 5. Univariable Cox Regression Analysis of Age Categories and 30-Day Stroke**

**eTable 6. Univariable Cox Regression Analysis of SVG Burden and 30-Day Stroke**

**eTable 7. Apparent Calibration of the AMP Prediction Model for 30-Day Stroke**

**eFigure 1. Calibration Plot for the AMP Prediction Model**

**eTable 1. Full Multivariable Cox Model Including AMP Score (Per 1-SD Increase)**

**Stratified by participating centers**

**N = 6,818
Events = 133**

| **Variable** | **Hazard Ratio (95% CI)** | **P value** |
| --- | --- | --- |
| **AMP_final (per 1-SD increase)** | 1.46 (1.23–1.73) | <0.001 |
| **Gender** | 0.62 (0.41–0.94) | 0.024 |
| **Hypertension** | 1.35 (0.91–1.99) | 0.131 |
| **Diabetes Mellitus** | 1.07 (0.76–1.52) | 0.692 |
| **Smoking** | 1.07 (0.75–1.53) | 0.712 |
| **COPD** | 1.52 (0.92–2.53) | 0.104 |
| **CKD** | 1.07 (0.64–1.79) | 0.798 |
| **Carotid Artery Stenosis (CAS)** | 1.09 (0.66–1.78) | 0.737 |
| **Cardiopulmonary Bypass (CPB) Time (per 10-minute increase** | 1.03 (0.97–1.08) | 0.088 |
| **LVEDD (per 1-mm increase)** | 1.01 (0.99–1.04) | 0.273 |
| **Septal thickness (per 1-mm increase)** | 1.04 (0.96–1.13) | 0.347 |
| **WBC (per 1-unit increase)** | 1.03 (0.98–1.08) | 0.259 |

**Model LR χ²(11) = 35.27
P = 0.0002**

**eTable 2. Full Multivariable Cox Model Including AMP Score (Per 1-SD Increase)**

**Stratified by participating centers**

**N = 6,818
Events = 133**

| **Variable** | **Hazard Ratio (95% CI)** | **P value** |
| --- | --- | --- |
| **AMP_final (per 2-unit increase)** | 1.62 (1.30–2.01) | <0.001 |
| **Gender** | 0.62 (0.41–0.94) | 0.024 |
| **Hypertension** | 1.35 (0.91–1.99) | 0.131 |
| **Diabetes Mellitus** | 1.07 (0.76–1.52) | 0.692 |
| **Smoking** | 1.07 (0.75–1.53) | 0.712 |
| **COPD** | 1.52 (0.92–2.53) | 0.104 |
| **CKD** | 1.07 (0.64–1.79) | 0.798 |
| **Carotid Artery Stenosis (CAS)** | 1.09 (0.66–1.78) | 0.737 |
| **Cardiopulmonary Bypass (CPB) Time (per 10-minute increase** | 1.04 (0.99–1.10) | 0.091 |
| **LVEDD (per 1-mm increase)** | 1.01 (0.99–1.04) | 0.273 |
| **Septal thickness (per 1-mm increase)** | 1.04 (0.96–1.13) | 0.347 |
| **WBC (per 1-unit increase)** | 1.03 (0.98–1.08) | 0.259 |

**Model LR χ²(11) = 35.27
P = 0.0002**

**eTable 3. Sensitivity Analysis Excluding Postoperative Atrial Fibrillation**

**Stratified by participating centers
N = 6,818
Events = 133**

| **Variable** | **Hazard Ratio (95% CI)** | **P value** |
| --- | --- | --- |
| **Adjusted (excluding postoperative AF)** |  |  |
| AMP_final (per 1-SD increase) | 1.46 (1.23–1.73) | <0.001 |
| AMP_final (per 2-unit increase) | 1.62 (1.30–2.01) | <0.001 |
| **Bootstrap adjusted (excluding postoperative AF)** |  |  |
| AMP_final (per 1-SD increase) | 1.46 (1.24–1.72) | <0.001 |
| AMP_final (per 2-unit increase) | 1.62 (1.31–1.99) | <0.001 |

**Models adjusted for sex, hypertension, diabetes mellitus, smoking status, chronic obstructive pulmonary disease (COPD), chronic kidney disease (CKD), carotid artery stenosis (CAS), Cardiopulmonary Bypass (CPB) Time, left ventricular end-diastolic diameter (LVEDD), interventricular septal thickness, and white blood cell (WBC) count. All Cox models were stratified by participating center. Bootstrap validation was performed using 1000 resamples.**

**eTable 4. Sensitivity Analysis Including Preoperative Atrial Fibrillation
Stratified by participating centers
N = 6,818
Events = 133**

| **Model** | **Variable** | **Hazard Ratio (95% CI)** | **P value** |
| --- | --- | --- | --- |
| Adjusted including preoperative AF | AMP_final (per 1-SD increase) | 1.47 (1.24–1.75) | <0.001 |
| Adjusted including preoperative AF | AMP_final (per 2-unit increase) | 1.63 (1.31–2.03) | <0.001 |
| Adjusted including preoperative AF | Preoperative atrial fibrillation | 2.87 (1.92–4.30) | <0.001 |
| Bootstrap adjusted including preoperative AF | AMP_final (per 1-SD increase) | 1.47 (1.25–1.73) | <0.001 |
| Bootstrap adjusted including preoperative AF | AMP_final (per 2-unit increase) | 1.63 (1.32–2.01) | <0.001 |
| Bootstrap adjusted including preoperative AF | Preoperative atrial fibrillation | 2.87 (1.91–4.31) | <0.001 |

**Models adjusted for sex, hypertension, diabetes mellitus, smoking status, chronic obstructive pulmonary disease (COPD), chronic kidney disease (CKD), preoperative atrial fibrillation, cardiopulmonary bypass (CPB) time, carotid artery stenosis (CAS), left ventricular end-diastolic diameter (LVEDD), interventricular septal thickness, and white blood cell (WBC) count. All Cox models were stratified by participating center. Bootstrap validation was performed using 1000 resamples.**

**eTable 5. Univariable Cox Regression Analysis of Age Categories and 30-Day Stroke**

| **Age category** | **Total patients, n** | **Stroke events, n (%)** | **Hazard ratio (95% CI)** | **P value** |
| --- | --- | --- | --- | --- |
| <60 years | 1,883 | 29 (1.54%) | Reference | — |
| 60–69 years | 2,790 | 49 (1.76%) | 1.14 (0.72–1.81) | 0.567 |
| 70–79 years | 1,833 | 45 (2.45%) | 1.63 (1.02–2.60) | 0.040 |
| ≥80 years | 312 | 10 (3.21%) | 1.86 (0.91–3.82) | 0.091 |

**Univariable Cox proportional hazards regression was used to evaluate the association between decade-based age categories and 30-day stroke. Patients aged <60 years were used as the reference group. The model was stratified by participating center. Overall model likelihood-ratio χ²(3) = 6.26; p = 0.0997.**

**eTable 6. Univariable Cox Regression Analysis of SVG Burden and 30-Day Stroke**

| **SVG category** | **Total patients, n** | **Stroke events, n (%)** | **Hazard ratio (95% CI)** | **P value** |
| --- | --- | --- | --- | --- |
| 0 SVG | 1,911 | 25 (1.31%) | Reference | — |
| 1 SVG | 3,222 | 60 (1.86%) | 1.72 (1.07–2.77) | 0.025 |
| 2 SVGs | 1,460 | 36 (2.47%) | 2.26 (1.35–3.78) | 0.002 |
| ≥3 SVGs | 225 | 12 (5.33%) | 3.64 (1.83–7.26) | <0.001 |

**Additional binary analysis:**

| **SVG category** | **Total patients, n** | **Stroke events, n (%)** | **Hazard ratio (95% CI)** | **P value** |
| --- | --- | --- | --- | --- |
| <3 SVGs | 6,593 | 121 (1.84%) | Reference | — |
| ≥3 SVGs | 225 | 12 (5.33%) | 2.32 (1.28–4.21) | 0.006 |

**Univariable Cox proportional hazards regression was used to evaluate the association between SVG burden and 30-day stroke. Models were stratified by participating center. For the categorical analysis, patients with 0 SVGs were used as the reference group. For the binary analysis, patients with <3 SVGs were used as the reference group.**

**eTable 7. Apparent Calibration of the AMP Prediction Model for 30-Day Stroke**

**A. Calibration performance metrics**

| **Calibration metric** | **Estimate** |
| --- | --- |
| Hosmer–Lemeshow χ² | 8.23 |
| Hosmer–Lemeshow p value | 0.4117 |
| Calibration-in-the-large | 0.00 |
| Calibration slope | 1.00 |
| Number of observations | 6,818 |
| Number of stroke events | 133 |

**B. Observed versus predicted 30-day stroke risk across deciles of predicted probability**

| **Decile of predicted risk** | **Patients, n** | **Stroke events, n** | **Mean predicted risk, %** | **Observed stroke risk, %** |
| --- | --- | --- | --- | --- |
| 1 | 682 | 2 | 0.47 | 0.29 |
| 2 | 682 | 4 | 0.69 | 0.59 |
| 3 | 682 | 6 | 0.86 | 0.88 |
| 4 | 682 | 10 | 1.03 | 1.47 |
| 5 | 681 | 6 | 1.22 | 0.88 |
| 6 | 682 | 10 | 1.46 | 1.47 |
| 7 | 682 | 6 | 1.80 | 0.88 |
| 8 | 682 | 22 | 2.33 | 3.23 |
| 9 | 682 | 22 | 3.29 | 3.23 |
| 10 | 681 | 45 | 6.36 | 6.61 |

**Calibration was assessed using predicted probabilities from the AMP logistic prediction model including AMP score, prespecified clinical covariates, and center indicators. Calibration-in-the-large and calibration slope represent apparent calibration within the derivation cohort. The Hosmer–Lemeshow test was performed using deciles of predicted risk.**

**eFigure 1. Calibration Plot for the AMP Prediction Model**

Calibration plot comparing observed 30-day stroke risk with mean predicted risk across deciles of predicted probability for the AMP prediction model. Points represent observed event rates within each decile of predicted risk, and the diagonal line represents perfect calibration. The plot demonstrates acceptable apparent calibration within the derivation cohort, with observed risk generally increasing across predicted-risk deciles.
